# Brain Cytoplasmic RNAs in Neurons: From Biosynthesis to Function

**DOI:** 10.3390/biom10020313

**Published:** 2020-02-17

**Authors:** Younghoon Lee, Hee-Seung Lee, Meehyein Kim, Heegwon Shin

**Affiliations:** 1 Department of Chemistry, Korea Advanced Institute of Science and Technology (KAIST), Daejeon 34141, Korea; Younghoon.Lee@kaist.ac.kr (Y.L.); hee-seung_lee@kaist.ac.kr (H.-S.L.); 2 Center for Multiscale Chiral Architectures (CMCA), Daejeon 34141, Korea; 3 Innovative Target Research Center, Korea Research Institute of Chemical Technology, Daejeon 34114, Korea; mkim@krict.re.kr

**Keywords:** noncoding RNA, brain cytoplasmic RNAs, neuron, translation inhibition, neuronal plasticity

## Abstract

Flexibility in signal transmission is essential for high-level brain function. This flexibility is achieved through strict spatial and temporal control of gene expression in neurons. Given the key regulatory roles of a variety of noncoding RNAs (ncRNAs) in neurons, studying neuron-specific ncRNAs provides an important basis for understanding molecular principles of brain function. This approach will have wide use in understanding the pathogenesis of brain diseases and in the development of therapeutic agents in the future. Brain cytoplasmic RNAs (BC RNAs) are a leading paradigm for research on neuronal ncRNAs. Since the first confirmation of brain-specific expression of BC RNAs in 1982, their investigation has been an area of active research. In this review, we summarize key studies on the characteristics and functions of BC RNAs in neurons.

## 1. Introduction

Prior to completion of sequencing of the human genome, the complexity of living organisms had been assumed to derive from the diversity of protein-coding genes, which were estimated to number ≈120,000 in the human genome [1,2]. The Human Genome Project, however, revealed the shocking finding that only ≈2% of the human genome encodes proteins [3]. The amount of noncoding DNA was much greater than had previously been predicted, and could no longer be treated as “junk DNA”. This realization prompted follow-up studies that sought to determine the biological significance of noncoding DNA. Subsequent extensive transcriptome analyses have established that the human genome is pervasively transcribed, with more than 60% of the genome producing RNAs [4,5,6,7].

Noncoding RNAs (ncRNAs), commonly defined as transcripts that lack the ability to produce a protein, are classified according to size as long noncoding RNAs (lncRNAs; >200 nucleotides (nt)) and small noncoding RNAs (sncRNAs; <200 nt). About 60,000 lncRNAs have been identified in human cells; as an indication that ncRNA research is still in its infancy, ≈70% of these lncRNAs remain unnamed [2,8]. Although these RNAs were initially thought to be transcription byproducts without specific functions [9,10], recent studies have shown that RNAs themselves or their transcription actively contribute to biological phenomena through a variety of mechanisms [8,11,12,13,14,15].

The brain, the most complex organ in the body, interprets a variety of stimuli from the outside world, generating the appropriate signal and passing it on to other organs. This process, carried out by 80 billion neurons spread throughout the central nervous system [16], owes much to the operation of networks of ncRNAs. Brain activity at the cellular level can be interpreted as a specific combination of neuronal activation and inactivation [17,18]. Again, at the molecular level, this can be expressed as a change in the balance between activation and inactivation of the expression of specific genes in neurons. For normal brain activity, this process must be signal-dependent and requires the highest-level expression control system [19,20,21,22,23]. Neurons utilize a variety of ncRNAs to achieve this [20,23,24,25,26]. In 2008, Mercer confirmed the expression of 849 ncRNAs in the adult mouse brain, many of which exhibited characteristic location patterns [27]. The hypothesis that all these ncRNAs have a definite function may be an exaggerated idea [28], but the research that reveals the meaning of their existence is essential for a deeper understanding of the brain, a complex organ.

In 1982, Sutcliffe and colleagues first discovered a polymerase III (pol III)-dependent ncRNA that was expressed only in the rat brain [29]. While analyzing poly A+ transcripts in the brain, liver, and kidney tissues, they found a small (152 nt) transcript that was specifically expressed in the brain and named it brain cytoplasmic 1 RNA (BC1 RNA) [29,30]. A subsequent study in 1987 identified a 200 nt-long RNA expressed in the primate brain that had common expression characteristics with BC1 RNA, naming it brain cytoplasmic 200 RNA (BC200 RNA) [31].

**Figure 1:**
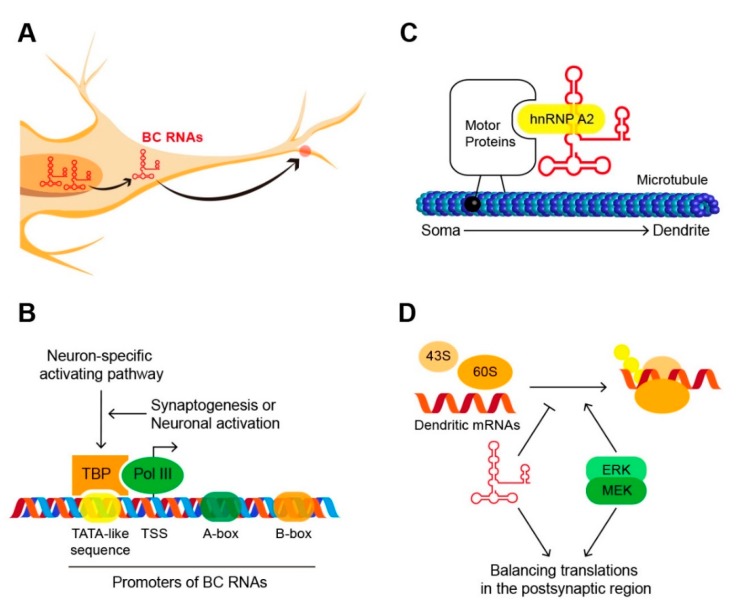
Brain cytoplasmic RNAs (BC RNAs) in neurons. (**A**) The journey of BC RNAs in neurons. (**B**) Promoters of BC RNAs consist of the core motif, TATA-like sequence in the upstream sequence and A-box and B-box in the internal sequence. TATA-binding protein (TBP) recognizes promoter sequences and recruits the polymerase III complex. These processes are regulated by sophisticated neuronal-specific activating pathway. (**C**) BC RNAs bind with heterogeneous ribonucleoprotein A2 (hnRNP A2) in the soma. RNP complex is migrated into dendrite along the microtubule. (**D**) Mitogen-activated protein kinase kinase/Extracellular signal-regulated kinase (MEK/ERK) promotes the translation of dendritic mRNA and BC RNAs inhibit excessive translation. BC RNAs are key factors maintaining translation balance in the postsynaptic region.

BC1 RNA and BC200 RNA, collectively called brain cytoplasmic RNAs (BC RNAs), are specifically located in neuronal dendrites and control protein translation by inhibiting translation–initiation factors (Figure 1) [32,33]. This regulation plays an important role in maintaining the appropriate protein translation balance in post-synaptic regions. The association of some neurological diseases with BC RNAs illustrates how important regulation by BC RNAs is for normal brain activity [34]. This review summarizes key studies of BC RNAs in neurons and may provide important insights into future research on ncRNAs in the brain—the subject of considerable recent attention.

## 2. BC RNAs: BC1 and BC200 RNA

In 1984, Sutcliffe and colleagues extended their initial work, examining BC1 RNA expression in additional tissues of the rat, including the adrenal gland, spleen, testis, lung, heart, muscle, and gut. At this point, BC1 RNA had still been identified only in the brain [30], and was thought to be a transcriptional byproduct of mRNA splicing because it was too short to function as mRNA. However, it was identified as an ncRNA because the corresponding DNA in the genome contained its own promoter and the ncRNA itself was located in the cytoplasm. In 1987, Dechiara and Brosius first sequenced BC1 RNA by generating complete cDNA [35]. They showed that BC1 RNA is transcribed from a single gene derived from a retroposed transfer RNA^Ala^ (tRNA^Ala^) harboring 3′ unique sequence. Their subsequent study confirmed that BC1 RNA is conserved among rodents, including the guinea pig, Syrian golden hamster, and mouse [36].

**Figure 2:**
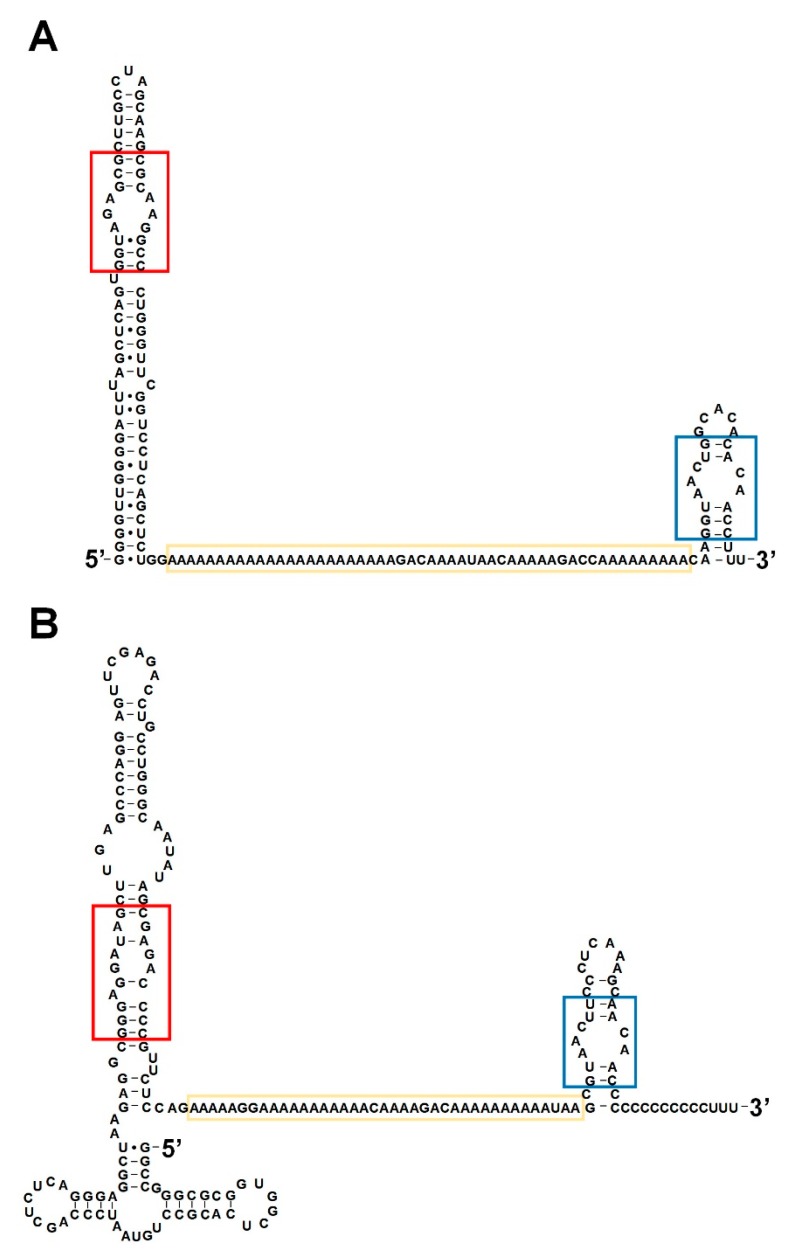
Secondary structures of BC1 RNA and BC200 RNA. (**A**,**B**) BC1 RNA and BC200 RNA are functional analogues. Secondary structures of BC1 RNA (**A**) and BC200 RNA (**B**) with common functional motifs (the GA kink motif in the red box, the poly A region in the yellow box, and the C-loop motif in the blue box).

In 1987, Watson and Sutcliffe identified a 200 nt-long RNA that exhibited brain-specific expression in primates and named it BC200 RNA. This gene is derived from a monomeric Alu element and the transcript has a poly A region similar to BC1 RNA, but the overall sequences are very different [31,36]. However, research over the past three decades has confirmed that BC1 and BC200 RNA are functional analogues that perform the same function in each neuron through key common motifs including the GA kink motif, the poly A region, and the C-loop motif (Figure 2) [32,37,38,39].

## 3. Biosynthesis and RNA Stability

In 2007, Khanam et al. showed in transgenic mice that the BC200 RNA gene harboring only 250 bp upstream sequences expressed a stable amount of RNA comparable to a construct harboring about 2.3 kb upstream [40]. In 2017, Kim et al. conducted a serial upstream deletion analysis to identify the promoter. They found that TATA-binding protein (TBP) binds to the position 28 to 22 upstream sequences, recruiting RNA polymerase III complex to initiate transcription. Additionally, they further confirmed the presence of an internal promoter in the 5′ terminal region of the transcript [41]. The BC1 RNA gene similarly has two types of promoters in the upstream and internal regions [42].

Normally, BC RNAs are expressed only in neurons, but this regulated expression disappears completely during tumorigenesis. In 1997, Chen et al. confirmed that BC200 RNA was abnormally expressed in esophageal, lung, breast, colon, and cervical cancer tissues [43]. Since then, studies have been conducted to analyze the causes of such abnormal expression. A study by Singh et al. showed that BC200 RNA expression is promoted in an estrogen receptor-dependent manner in breast cancer cell lines [44]. Using chromatin immunoprecipitation (ChIP) assays, these authors confirmed that estrogen receptor-α binds directly to the upstream region of BC200 RNA to promote transcription. Another study by Hu and Lu confirmed that BC200 RNA is induced by an oncogenic transcriptional factor, c-MYC in non-small cell lung cancer cells [45]. However, it is not yet known whether these factors and mechanisms are involved in the expression of BC RNAs in neurons.

Recently, Sonawane and his colleagues published important results on tissue-specific gene expression [46]. They collected expression information for 30,243 proteins from 38 tissues and analyzed the regulatory network pattern in each tissue by incorporating the initial regulatory network information for 644 transcription factors and transcription factor protein–protein interactions [47,48]. They found that tissue-specific expression is determined by tissue-specific regulatory network pathways rather than by the expression of specific transcription factors. This finding shows that identifying the neuron-specific regulatory network that controls ncRNA expression is necessary to further understand the biosynthesis of BC RNAs.

Another characteristic of the biosynthesis of BC RNAs is that the level of expression is strongly affected by the development and activity of neurons. In 1998, Muslimov et al. observed that BC1 RNA remained low until synapses were fully formed and that its expression was suppressed by neuronal activity inhibitor, tetrodotoxin [49]. Activity-dependent expression provided an important clue for the functional significance of BC RNAs in neurons.

RNA stability is another factor that determines the number of intracellular transcripts [50]. Kim et al. compared the stability of BC200 RNA in various cancer cell lines and showed that the half-life of BC200 RNA changes significantly depending on the cell line [41]. This indicates that the environment in which BC RNA exists varies greatly from cell to cell.

## 4. Functions of BC RNAs

BC RNAs are strong translational regulators. In 2002, using an in vitro system, Wang and colleagues first discovered that BC1 RNA exerts a translation-inhibitory effect [51]. Subsequence studies confirmed that BC1 RNA binds to eukaryotic initiation factor 4A (eIF4A) and poly A binding protein (PABP), and inhibits cap-dependent and internal ribosome entry site (IRES)-dependent translation [52,53]. eIF4A is an ATP-dependent RNA helicase that initiates translation by unwinding the structure of the 5′ mRNA [54]. In 2008, Lin et al. found that BC1 RNA extinguishes the molecular force of eIF4A obtained through ATP hydrolysis. This was the first confirmation that BC1 RNA directly inhibits the activity of a single enzyme [55]. BC RNAs were also found to regulate translation in a manner that inhibits the function of eIF4B [56,57].

The generation of BC1-knockout (BC1-KO) mice by Skryabin et al. was an important starting point for the physiological study of BC RNAs [58]. In 2004, Lewejohann and colleagues found that BC1-KO mice have behavioral changes, such as reduced exploration, increased anxiety, and reduced survival in outdoor environments [59]. Independent studies by Zhong and Maccarrone confirmed abnormal increases in transmission that depended on metabotropic glutamate receptors (mGluRs) in BC1-KO mice [60,61]. In 2017, Briz et al. confirmed that a BC1 deficiency causes expression imbalances in the postsynaptic region, promoting an abnormal expression of glutamate receptor subunits that results in excessive signal transmission. The resulting hyperexcitation of neurons extinguishes synaptic plasticity, leading to severe defects in cognition and learning [62].

Taken together, these observations indicate that BC RNAs regulate signal transmission by maintaining a balance between translational activation and inhibition in postsynaptic regions. This regulation allows neurons to exhibit experience- and context-dependent plasticity and ultimately perform higher-level brain functions, such as cognition and learning.

## 5. Regulatory Factors

Although BC RNAs can affect global expression in vitro, they may have a limited effect in vivo [53,63]. This suggests the presence of higher regulators that control the functions of BC RNAs in cells. The specific localizing of BC RNA in neurons is one of the well-studied mechanisms of BC RNA activity control [64]. Neurons are structurally and functionally asymmetrical cells, and thus the location of the components is finely adjusted according to their function [65,66]. BC RNAs are preferentially localized in the dendrite through the GA kink-turn motif in the 5′ stem-loop [51,64,67,68,69]. This is a common structural characteristic of dendritic mRNAs and is recognized by heterogeneous ribonucleoprotein A2 (hnRNP A2), leading to microtubule-dependent dendritic localization of RNAs [68,69,70]. This mechanism limits the activity of BC RNAs to the microenvironment within the neuronal dendrites.

In 2019, a study by Muslimov et al. confirmed that the dendritic localization of BC200 RNA was severely impaired in the autoimmune disease systemic lupus erythematosus (SLE) [38]. They further detected specific antibodies against BC200 RNA in the serum of SLE patients and found that the antibody competitively inhibited binding to hnRNP A2. These findings show how important the dendritic localization of BC200 RNA is for normal brain activity.

In 2003, Zalfa and colleagues reported that BC1 RNA binds to fragile X mental retardation protein (FMRP), suggesting that the BC1 RNA-FMRP complex might regulate the translation of specific mRNAs [71]. However, in 2008, Iacoangeli et al. revealed that BC1-FMRP binding affinity is as low as the tRNA-FMRP level and there is no binding in vivo either [72]. These verification experiments led to the conclusion that the interaction between BC1 and FMRP is merely nonspecific binding. The recent results of Booy et al. also support this conclusion [73]. Their analysis of proteins interacting with BC RNAs at the genome level showed binding to 14 proteins including 8 new proteins, but no direct binding to FMRP.

Booy et al.’s work offers potential for new regulatory systems of BC RNA function in neurons [73]. Among newly discovered binding proteins, poly C binding protein 2 (PCBP2) has been shown to control the translational inhibition of BC200 RNA in vitro [74]. Further research is needed to see whether other interactions have regulatory effects and whether these regulators also work in real neurons.

## 6. Neuronal Diseases Related to BC RNAs

BC RNAs are associated with several neuronal diseases (Table 1). In 2007, Mus and colleagues reported that BC200 RNA was associated with Alzheimer’s disease (AD) [34]. They investigated BC200 RNA expression levels in Brodmann’s area 9, known to be severely affected in AD, at different ages. They found that, in a normal brain, expression of BC200 RNA gradually decreases with age. However, they confirmed that BC200 RNA levels were increased by ≈2-fold in the brains of AD patients of a similar age. The degree of abnormal expression increased in proportion to the clinical dementia rating. Additionally, BC200 RNAs in patients’ brains were abnormally distributed, showing high-intensity clusters in the soma. However, how BC200 RNA expression is promoted in AD and how it affects the pathogenesis of AD are not known.

Fragile X syndrome (FXS) is a congenital brain disease characterized by a wide range of neuropsychiatric manifestations, including mental retardation, hyperactivity, and autism [75]. The main cause is decreased expression of FMRP protein owing to a mutation in the *FMR1* gene on the X chromosome [76]. Independently of BC RNAs, FMRP contributes to building synaptic plasticity by regulating translation of dendritic mRNAs [77]. Although normal people have between 6–54 CGG repeats in the *FMR1* gene, FXS patients have over 200 CGG repeats, which reduces the expression of FMRP protein and causes brain dysfunction [78]. BC1-KO mice show abnormal neuronal transmission patterns that are similar to those of FMRP-KO mice [61,79], suggesting that the loss of the BC RNAs function may have a similar pathological effect.

In addition, CGG repeats of the *FMR1* gene have been reported to directly affect the function of BC RNAs. People with 55-200 CGG repeats inserted into the *FMR1* gene are classified as premutation [78]. Young premutation carriers may present with cognitive disturbances and aged carriers may develop fragile X-associated tremor/ataxia syndrome (FXTAS), a neurodegenerative disorder; however, detailed pathogenesis is not yet well understood [80]. Muslimov et al. established and analyzed CGG knock-in mice [81]. They found that most of the BC1 RNA remained near the nucleus in neurons and confirmed that these neurons were hyper-excited by signaling and caused cognitive decline. CGG repeat motif strongly bound to hnRNP A2, inhibiting the localization of BC RNAs by weakening the interaction between hnRNP A2-BC RNAs. Through these observations, they suggested that the mislocalization of BC RNAs by CGG repeats could be the cause of fragile X premutation disorders.

Muslimov and colleagues recently reported that the mislocalization of BC RNAs is also associated with SLE [38]. Researchers confirmed the production of antibodies that recognize BC200 RNA in SLE patients and named them anti-BC abs. They found that anti-BC abs are not detectable in normal cells or in other autoimmune diseases. Anti-BC abs bind to the 5′ stem-loop of BC200 RNA and inhibit the binding of hnRNP A2, resulting in a defect in dendritic localization. Finally, they confirmed that administration of anti-BC abs to normal mice resulted in phenotypic defects, such as epileptic-induced responses and impaired cognitive function.

## 7. BC RNAs in Cancers

In 1997, abnormal expression of BC200 RNA was first detected in several cancer tissues, including breast, esophagus, lung, ovary, parathyroid, and tongue [43]. In 2004, more invasive cancer cells were found to express higher levels of BC200 RNA, showing the possibility of contributing to cancer development [82]. The detailed mechanism had not been well understood for a long time, but has recently begun to be proposed by some studies [44,63,83].

In 2016, Singh and colleagues constructed BC200 knock-out cell lines by the clustered regularly interspaced short palindromic repeats/CRISPR associated protein 9 (CRISPR/Cas9) system [44]. They observed that the deletion of the BC200 gene inhibits cell growth by activating the apoptosis of the cancer cells. They showed that BC200 RNA partially binds to B-cell lymphoma-extra (Bcl-x) mRNA, inhibiting splicing to Bcl-xS, the apoptosis-promoting factor. However, further studies are required that verifythe real interaction between BC200 RNA and Bcl-x mRNA in vivo and show how cancer BC RNAs, mainly located in the cytoplasm in neurons, work in the nucleus in cancer cells.

In 2017, Shin et al. investigated the effect of BC200 RNA on genome-wide expression profiling of the cervical cancer cell line HeLa [63]. As a result, they found that expressions of 29 genes are altered by BC200 RNA knockdown. Among them, the expression of S100A11, previously identified as the cell mobility activating factor, was significantly reduced. The researchers showed that BC200 RNA promotes cell mobility of HeLa cells by stabilizing S100A11 mRNA and promoting its expression. However, it is not yet known how BC200 RNA enhances the stability of S100A11 mRNA. Additionally, some studies suggest that BC200 RNA may promote the development of cancer cells by inhibiting tumor-suppressive miRNAs [84,85].

The study of BC RNAs in cancer has a relatively shorter history. Although more detailed research on mechanisms is required, it is interesting to have the potential shown for BC200 RNA to act as a more diverse mechanism within cells compared to previous studies in neurons. Future studies need to confirm whether these mechanisms are also preserved in neurons. Although the environment of BC RNAs varies dramatically from cell to cell, neurons and cancer cells may share some of the same binding proteins and functions of BC RNAs. The BC RNAs of neurons have the potential to regulate gene expression through more complex mechanisms than have been identified to date.

## 8. Conclusions

If a protein is the object that performs a physical function in a cell, ncRNAs are regulators that control this function so that it occurs at the right time and place [11]. The proportion of ncRNAs in the genome increases as biological complexity increases [86], suggesting that the higher-order functions of complex organisms may be more dependent on the number of ncRNAs than on the number of proteins. The human brain performs the highest-level functions of all organs in the body. The process of recognizing, interpreting, and responding to the diverse situations encountered requires the most sophisticated control systems in all living organisms. It is known that various classes of neuronal ncRNAs actively contribute to this regulation [20,64,87,88,89].

**Figure 3:**
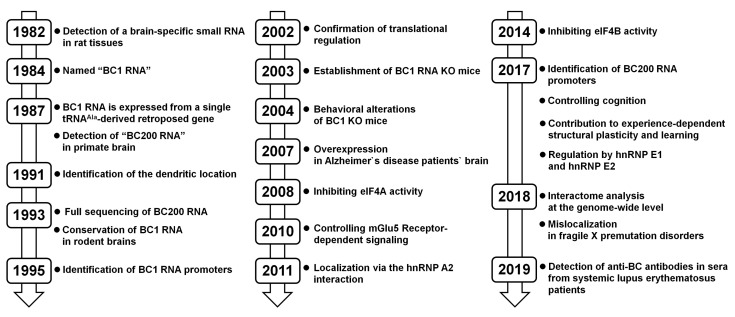
History of BC RNA studies in neurons.

The long history of BC RNA research in the neuron is divided into three stages (Figure 3). In the first stage, early research examined the expression characteristics of BC RNAs. RNA expression analyses of each tissue revealed that BC1 RNA was specifically expressed in rat brain and BC200 RNA was expressed in the human brain. In the second stage, in vitro and cell-based experiments revealed the molecular function of BC RNAs in neurons. The binding partners of BC RNAs were identified and their interactions were shown to exert potent translation-inhibitory functions in post-synaptic regions. In the third stage, the physiological functions of BC RNAs in neurons were explored. Behavioral experiments using BC1-KO mice showed that BC RNAs are involved in higher-level brain functions, such as learning and cognition. Combined with molecular-level functional studies, this research showed that BC RNAs play an important role in the formation of neuronal plasticity involved in signal-dependent activation, while maintaining a translational balance in dendrite regions.

This flow of studies provides important inspiration for future research of BC RNAs and other ncRNAs in neurons. An enormous number of ncRNAs expressed in the central nervous system have still remained in the first stage [24,27,90]. Multifaceted functional analyses of neuron-specific transcripts are needed. To this end, an analysis of genome-wide effects is essential, and it will be necessary to establish animal models for the analysis of physiological functions. Recent advances in sequencing analysis and genome editing technology using CRISPR/Cas9 are accelerating these studies.

## Figures and Tables

**Table 1:** Neuronal diseases associated with BC RNAs.

Disease	BC RNAs	Description
Alzheimer’s Disease	Up-regulation	Overexpressed in Brodmann’s area 9 and accumulated in the soma
Fragile X Syndrome	Down-regulation	BC1-KO mice are similar to FMRP-KO mice
Fragile X Premutation Disorders	Mislocalization	CGG repeats inhibits dendritic localization of BC RNAs
Systemic Lupus Erythematosus	Mislocalization	Anti-BC antibodies inhibit dendritic localization of BC RNAs

## Abbreviations

ncRNA, noncoding RNA; BC RNA, brain cytoplasmic RNA; pol III, polymerase III; MEK/ERK, mitogen-activated protein kinase kinase/extracellular signal-regulated kinase; TBP, TATA-binding protein; ChIP, chromatin immunoprecipitation; eIF, eukaryotic initiation factor; PABP, poly A binding protein; IRES, internal ribosome entry site; KO, knock-out; mGluR, metabotropic glutamate receptor; hnRNP, heterogeneous ribonucleoprotein particle; FMRP, fragile X mental retardation protein; PCBP, poly C binding protein; AD, Alzheimer’s disease; FXS, fragile X syndrome; FXTAS, fragile X-associated tremor/ataxia syndrome; SLE, systemic lupus erythematosus; CRISPR/Cas9, clustered regularly interspaced short palindromic repeats/CRISPR associated protein 9; Bcl-x, B-cell lymphoma-extra
